# Activities of Digestive Enzymes in the Omnivorous Pest *Apolygus lucorum* (Hemiptera: Miridae)

**DOI:** 10.1093/jee/tow263

**Published:** 2016-12-29

**Authors:** Wenjing Li, Xincheng Zhao, Wei Yuan, Kongming Wu

**Affiliations:** 1 State Key Laboratory for Biology of Plant Diseases and Insect Pests, Institute of Plant Protection, Chinese Academy of Agricultural Sciences, Beijing 100193, China (liwenjingpingyu@163.com; yuanwei19890126@163.com; kmwu@ippcaas.cn); 2 College of Plant Protection, Henan Agricultural University, Zhengzhou 450002, China xincheng@henau.edu.cn

**Keywords:** *Apolygus lucorum*, digestive enzyme, salivary gland complex, midgut, diet

## Abstract

The mirid bug *Apolygus lucorum* Meyer-Dür, 1843, an omnivorous species that feeds on plants and animals, has become a major pest in China as production of Bt-cotton has grown to such a large scale. Its omnivory is likely to be critical for its success, but the digestive mechanism(s) underlying processing and adsorption of such diverse foods is relatively unknown. Here, we examined the activities of digestive enzymes of *A. lucorum* in the salivary gland complex and midgut and the effect of sex, age, and food source on these activities. Amylase and protease were present in the salivary gland complex and the midgut, but were higher in the salivary gland complex. Trypsin-like enzyme was also present in both organs, but chymotrypsin-like enzyme was present only in the midgut. Sex, age, and food source affected the activities of these digestive enzymes. In general, the activities of these enzymes peaked at 10 d after emergence, and amylase and protease activities were higher in female adults than in males. Of the food sources tested, green bean pods (Gb) induced the highest amylase activity, whereas *Helicoverpa armigera* Hübner, 1809 eggs (He) and a mixture of Gb and He induced higher activities of the trypsin-like and chymotrypsin-like enzymes. The results from food switching experiments confirmed that amylase activity could be induced by plant sources, and animal sources induced protease activity. Thus, the types and activities of digestive enzymes in *A. lucorum* provide the physiological basis of the pest’s omnivory.

The mirid bug *Apolygus lucorum* Meyer-Dür, 1843 (Hemiptera: Miridae) has become a major pest of cotton in conjunction with the large-scale adoption of Bt-cotton (genetically modified with the *Cry1Ac* gene from *Bacillus thuringiensis*) in recent decades in China (Lu et al. 2010). The omnivory of *A. lucorum* likely contributed to its outbreak. In addition to plants, it can digest small insects; for example, it preys on eggs of *Helicoverpa armigera* Hübner, 1809 (Lepidoptera: Noctuidae), nymphs of *Aphis gossypii* Glover (Hemiptera: Aphididae) and *Bemisia tabaci* (Gennadius) (Hemiptera: Aleyrodidae) (Wang 2010, Li et al. 2016). Previous studies from our laboratory showed that a combination of plant and animal food sources facilitates the development of the ovary of *A. lucorum* females, and the ovaries have better-developed ovarioles with more previtellogenic, vitellogenic, and mature follicles (Yuan et al. 2013).

The nature of the food source, i.e., plant or animal, that an insect can assimilate is related to the type of digestive enzymes that the insect secretes into the salivary gland complex and the midgut of the insect, and the substrates used by these enzymes (Agusti and Cohen 2000, Torres and Boyd 2009). Herbivorous insects secrete more amylases (Cohen 1996), whereas carnivorous insects secrete greater amounts of proteases (Zeng and Cohen 2000, Boyd et al. 2002). The strictly phytophagous mirid *Poecilocapsus lineatus* F. (Heteroptera) lacks detectable digestive proteases (Cohen and Wheeler 1998), indicating that *P. lineatus* is unable to use animal protein. The omnivorous mirids *Lygus lineolaris* (Palisot de Beauvois) and *Lygushesperus* Knight have amylases and trypsin-like enzymes in their salivary gland complexes (Agusti and Cohen 2000, Wright et al. 2006), whereas the predatory mirid *Andrallus spinidens* F. has trypsin-like enzymes but not amylase in its salivary glands (Zibaee et al. 2012).

*Apolygus* *lucorum* can also feed on both plants and small insects. However, we have almost a complete lack of knowledge about the digestive physiology of this species. In this study, we firstly characterized the activities of digestive enzymes in the salivary gland complex and midgut of *A. lucorum*. Next, we examined the effects of sex, age, and food source on the activity of digestive enzymes of *A. lucorum*. The results of this study demonstrate that the types of digestive enzymes and their activities confer the digestive adaptations for zoophagy and phytophagy in *A. lucorum*.

## Materials and Methods

### Insect Rearing

Nymphs and adults of *A. lucorum*, which were collected from cotton fields at the Langfang Experimental Station of the Chinese Academy of Agricultural Sciences in Hebei Province in 2005, were used to establish a laboratory colony. The colony was infused every year with wild-type genotypes. The colony was maintained in a greenhouse on green bean pods, which served as a food and oviposition substrate for the mirids (Lu and Wu 2008), under standard environmental conditions (25 ± 2°C, 60 ± 5% relative humidity [RH], and a photoperiod of 14:10 [L:D] h). Individuals of *H. armigera* used for the diet were from a laboratory colony that was maintained at 27 ± 2°C, 75 ± 10% RH, and a photoperiod of 14:10 (L:D) h (Liang et al. 2008).

For the assays of the digestive enzymes of *A. lucorum* in both sexes at different ages, we excised the salivary gland complex and midgut separately from 1-, 5-, 10-, 15-, 20-, and 25-d-old male and female adults from the laboratory colony.

Because the digestive enzymes in *A. lucorum* originate in the salivary gland complex rather than the midgut, and because enzyme activities were greater in female adults than in male adults, the salivary gland complex from female adults was used in experiments on different foods and on food switching. For comparing the activities of digestive enzymes of *A. lucorum* reared on different foods, three types of food sources were used: 1) green bean pods (Gb) for the plant source, 2) a plant and an animal source together, green bean pods and *H. armigera* eggs (GbHe), and 3) the animal source alone, *H. armigera* eggs (He). Female adults that had fed on the Gb and GbHe diet at 1, 5, 10, 15, 20, and 25 d old and on the He diet at 1, 5, and 10 d old (most insects fed on the He diet alone died when 10 d old) were collected for dissecting salivary gland complex for digestive enzymes assay.

To test the effect of food source switching on the activities of digestive enzymes in *A. lucorum*, three food sources (Gb, GbHe, and He) were used. Five-day-old female adults that were reared on Gb, GbHe, or He since they had hatched from eggs were used for the experiments. Six treatments were designed: 1) Gb diet, then transfer to GbHe (Gb-to-GbHe); 2) Gb diet, then transfer to He (Gb-to-He); 3) GbHe diet, then transfer to Gb (GbHe-to-Gb); 4) GbHe diet, then transfer to He (GbHe-to-He); 5) He diet, then transfer to Gb (He-to-Gb); 6) He diet, then transfer to GbHe (He-to-GbHe). Those kept on the original food were used as controls. Female adults were collected at 1, 3, 5, 7, and 10 d after the transfer in the first four treatments for excising salivary gland complex; in the other two treatments, female adults were collected 1, 3, and 7 d after the transfer because the average longevity of female adults fed on the He diet was 10 d.

### Enzyme Sample Preparation

Enzyme samples were prepared using the method of Cohen (1993), with slight modifications. Adults of *A. lucorum* were starved for 12 h before dissection. The insects were dissected in ice-cold phosphate-buffered saline (PBS; pH 7.4) using a stereomicroscope. The salivary gland complex, including all lobes, accessory glands, and tubules, was exposed by removing the prothorax from the abdomen with fine forceps. The midgut was exposed by holding the body with the forceps and pulling the last three or four segments of the abdomen away from the rest of the abdomen with another pair of forceps. The salivary glands and midguts from *A. lucorum* were collected separately, then put in a cold homogenizer with PBS. Tissues were homogenized and then transferred to a 1.5-ml centrifuge tube and centrifuged at 12,000 rpm for 15 min at 4°C. The resulting supernatants were maintained at −80°C for further use. At least three biological replicates were assayed as triplicate technical replicates for each treatment, and each biological replicate was collected from 50 adults.

The protein concentrations of all of the enzyme samples were determined using the Bradford method with bovine serum albumin as a reference standard according to the manufacturer’s instructions (Bradford 1976).

### Enzyme Activity Assays

Amylase activity was assayed using the dinitrosalicylic acid (DNS) procedure (Bernfeld 1955) with 1% soluble starch (Merck, Darmstadt, Germany) as the substrate. Ten microliters of the enzyme sample was incubated for 30 min at 37°C with 500 μl universal buffer (40 mM sodium acetate-phosphate-borate) and 40 μl soluble starch. After incubation, the reaction was stopped with the addition of 100 μl DNS and heating in boiling water for 10 min, then cooled in ice for 5 min. After cooling, absorbance was read at 540 nm on an ELx808iu microtiter plate reader (Bio-Tek Instruments, Winooski, VT). A standard curve of absorbance against concentrations of maltose (Sigma Chemical Co., St. Louis, MO) released was constructed to calculate amylase activity units. One unit of amylase activity was defined as the amount of enzyme required to produce 1 μM maltose/min at 37°C under the given assay conditions.

The activity of trypsin-like enzymes was assayed as previously described (Zhu et al. 2003) with slight modifications. The substrate solution was prepared by dissolving *N*_α_-benzoyl-l-arginine-*p*-nitroanilide (BA*p*NA; B-3133, Sigma, St. Louis, MO) in 1 ml of dimethyl methyl sulfoxide before adding 49 ml of universal buffer (pH 7.5) to obtain a final BA*p*NA concentration of 1 mg/ml. Ten microliters of enzyme extract was mixed with 40 μl of universal buffer in a microtiter plate, and the mixture was incubated at 30°C for 20 min before 100 μl substrate was added. Absorbance was measured at 405 nm. A no-enzyme control was included to correct for endogenous hydrolysis of substrate. One unit of enzyme (U) is defined as the amount that hydrolyzes 1 μmol of substrate per minute. Mean enzyme activity were calculated from triplicate readings. The experiment was repeated with different enzyme preparations.

Chymotrypsin-like enzyme activity was assayed as previously described (Zibaee et al. 2012) with slight modifications. Substrate solution was prepared by dissolving *N*-succinyl-alanine-alanine-proline-phenylalanine-*p*-nitroanilide (SAAPP*p*NA; B-6760, Sigma) in 1 ml of dimethylformamide before adding 24 ml of universal buffer to obtain a final SAAPP*p*NA concentration of 1 mg/ml. Five microliters of enzyme sample was mixed with 90 μl of universal buffer in a microtiter plate, and the mixture was incubated at 30°C for 20 min before 45 μl substrate was added. Absorbance was measured at 405 nm. Chymotrypsin-like enzyme activity was calculated as done for trypsin-like enzyme activity. One unit of enzyme (U) was defined as the amount that hydrolyzes 1 μmol of substrate per minute.

### Statistical Analyses

Data were subjected to an analysis of variance (ANOVA), and the means were compared by a least square difference test (LSD). Statistical analyses were performed using the software Statistics Analysis System V8.

## Results

### Activities of Different Digestive Enzymes in *A. lucorum*

**Fig. 1.**
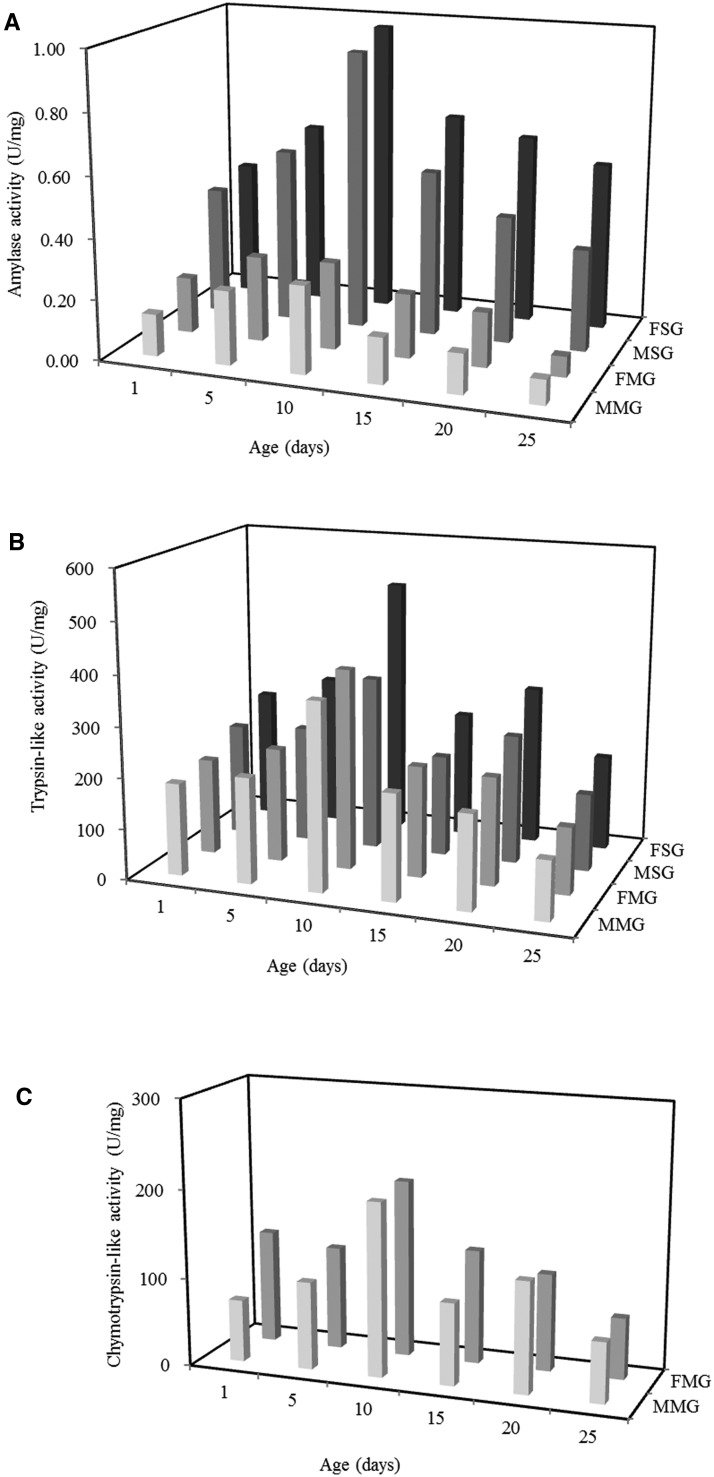
Mean activities of three types of digestive enzymes in the salivary gland and midgut of male and female *A. lucorum* at different ages (days after emergence). (**A**) Amylases; (**B**) Trypsin-like enzyme; (**C**) Chymotrypsin-like enzyme. FSG: female salivary gland; MSG: malesalivary gland; FMG: female midgut; MMG: male midgut.

Male and female *A. lucorum* adults secreted amylase in the salivary gland complex and midgut at different ages (Fig. 1A). In the salivary gland complex of both sexes, the activity of amylase remained lower in the early stage, and increased up to 10-d-old adult, then began to decline. Amylase activities in the salivary gland complex of females peaked (0.97 ± 0.10 U/mg protein) at 10 d of age. Amylase activities in the midgut were much lower than in the salivary gland complex, but varied similarly at different ages in both sexes.

Trypsin-like enzyme hydrolysis was detected in the enzyme extract from the salivary gland complex and midgut of males and females at different ages with BA*p*NA as the substrate (Fig. 1B). Trypsin-like enzyme activity in the salivary gland complex of females and males first increased, then decreased with increasing age, and peaked in 10-d-old insects. Activities in insects of the same age were generally higher in females than in males. Trypsin-like enzyme activity in the salivary gland complex was higher than in the midgut. The highest enzyme activity (507.61 ± 39.82 U/mg protein) was observed in the salivary gland complex in 10-d-old females.

Chymotrypsin-like enzyme activities measured in extracts of the salivary gland complex and midgut of *A. lucorum* at different ages are shown in Fig. 1C. The substrate SAAPP*p*NA was hydrolyzed by homogenates of midgut but not salivary gland. The activity of chymotrypsin-like enzyme was highest (200.89 ± 34.44 U/mg protein) in the midgut of 10-d-old females. Chymotrypsin-like enzyme activities in the midgut of female and male adult fluctuated with increasing age. The results showed that enzyme activities in the midgut were higher in females than in males of all ages except 20-d-old adults.

### Effects of Food Source on Digestive Enzyme Activities

**Fig. 2.**
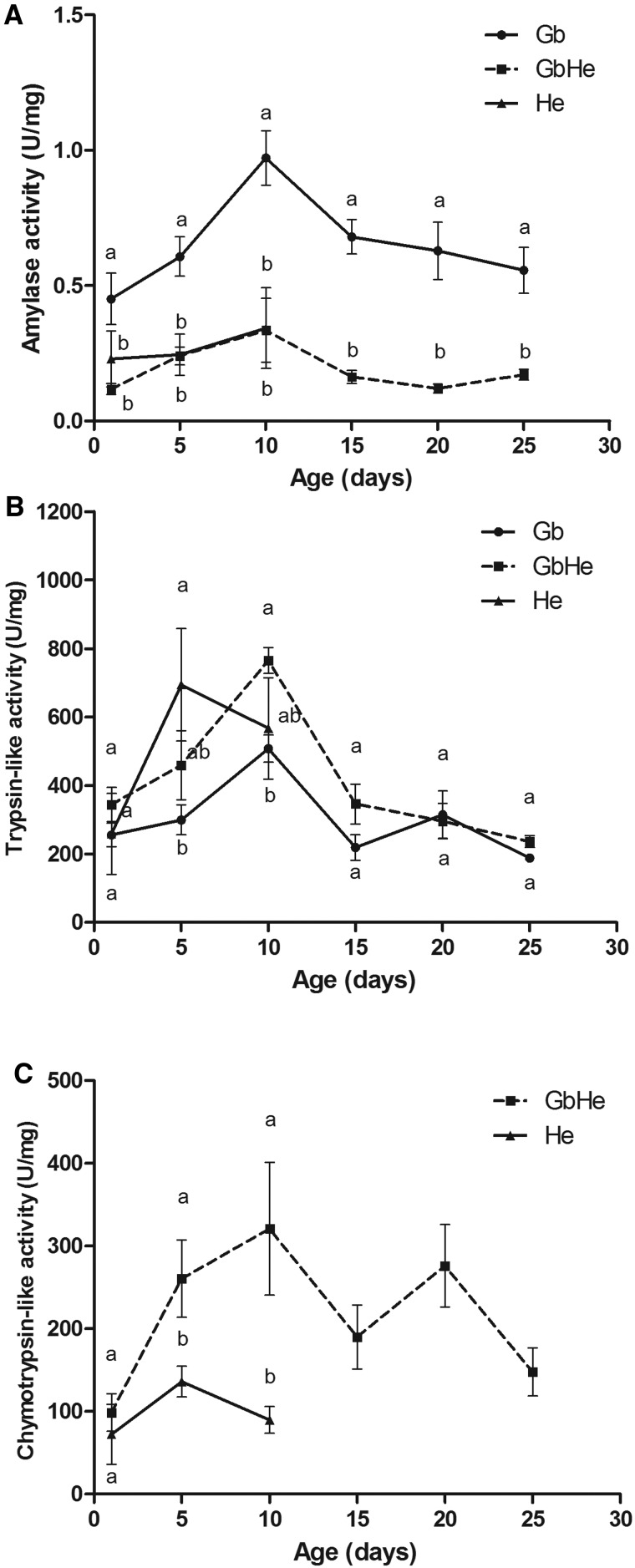
Activities of three types of digestive enzymes in the salivary gland of female *A. lucorum* fed on green beans (Gb), green beans and *H. armigera* eggs (GbHe), or *H. armigera* eggs (He). (**A**) Amylases; (**B**) Trypsin-like enzyme; (**C**) Chymotrypsin-like enzyme. Different letters by means for the same age among the various diets indicate significant differences at *P < *0.05 in a one-way ANOVA.

The activities of amylase, trypsin-like and chymotrypsin-like enzymes differed among food sources (Fig. 2). Gb-fed *A. lucorum* had higher amylase activities than those fed on He and GbHe. Protease activity (trypsin-like and chymotrypsin-like enzymes) in adults fed on He and GbHe was higher than in the Gb-fed adult.

*Apolygus* *lucorum* fed on Gb had higher amylase activity than those fed on He and GbHe (Fig. 2A). For the three types of food treatments, the activities of amylase in newly emerged adults were extremely low and first increased then decreased with increasing age. The maximum amylase activity in adults, regardless of diet, was reached at 10 d old. At 1, 5, and 10 d of age, the activity of amylase was significantly higher with the Gb diet than with GbHe and He (for 1-d-old female adults: *F*_2,10 _=_ _7.20, *P *= 0.012; for 5 d old: *F*_2,11 _=_ _12.14, *P *= 0.002; for 10 d old: *F*_2,11 _=_ _10.64, *P *= 0.003). In 15-, 20-, and 25-d-old female adults, amylase activity was significantly higher with the Gb diet than with GbHe (for 15-d-old female adults: *F*_1,9 _=_ _67.37, *P *= 0.001; for 20-d-old: *F*_1,7 _=_ _37.25, *P *= 0.001; for 25-d-old: *F*_1,5 _=_ _20.50, *P *= 0.006).

*Apolygus* *lucorum* on the Gb diet had lower trypsin-like enzyme activity than on the He or GbHe diet (Fig. 2B). The highest enzyme activity (765.19 ± 38.05 U/mg protein) was found in GbHe of 10-d-old female adults. In 1-d-old females, trypsin-like enzyme activity did not differ among the three diets (*F*_2,7 _=_ _0.53, *P *= 0.611). In insects 5 d old, trypsin-like enzyme activity with the He diet was significantly higher than with Gb, but did not differ from GbHe (*F*_2,8 _=_ _3.48, *P *= 0.082). In insects 10 d old, trypsin-like enzyme activity with GbHe was significantly higher than with Gb, but did not differ from He (*F*_2,8 _=_ _2.97, *P *= 0.109). In 15-, 20-, and 25-d-old adults, trypsin-like enzyme activities did not differ significantly between Gb and GbHe (for 15-d-old female adults: *F*_1,5 _=_ _2.86, *P *= 0.152; for 20-d-old: *F*_1,5 _=_ _0.05, *P *= 0.833; for 25-d-old: *F*_1,5 _=_ _5.12, *P *= 0.073).

*Apolygus* *lucorum* fed on He or GbHe had high activities of chymotrypsin-like enzyme but none on Gb treatment (Fig. 2C). With increasing age, the activity first increased and reached its peak at 10 d for GbHe and at 5 d for He, then decreased. Chymotrypsin-like enzyme activities were significantly higher after feeding GbHe than He at 5 and 10 d (for 5-d-old female adults: *F*_1,6 _=_ _6.10, *P *= 0.049; for 10-d-old females: *F*_1,6 _=_ _7.95, *P *= 0.030), but there was no significant difference at 1 d of age (*F*_1,6 _=_ _0.38, *P *= 0.562).

### Effects of Food Switching on Amylase Activities

**Fig. 3.**
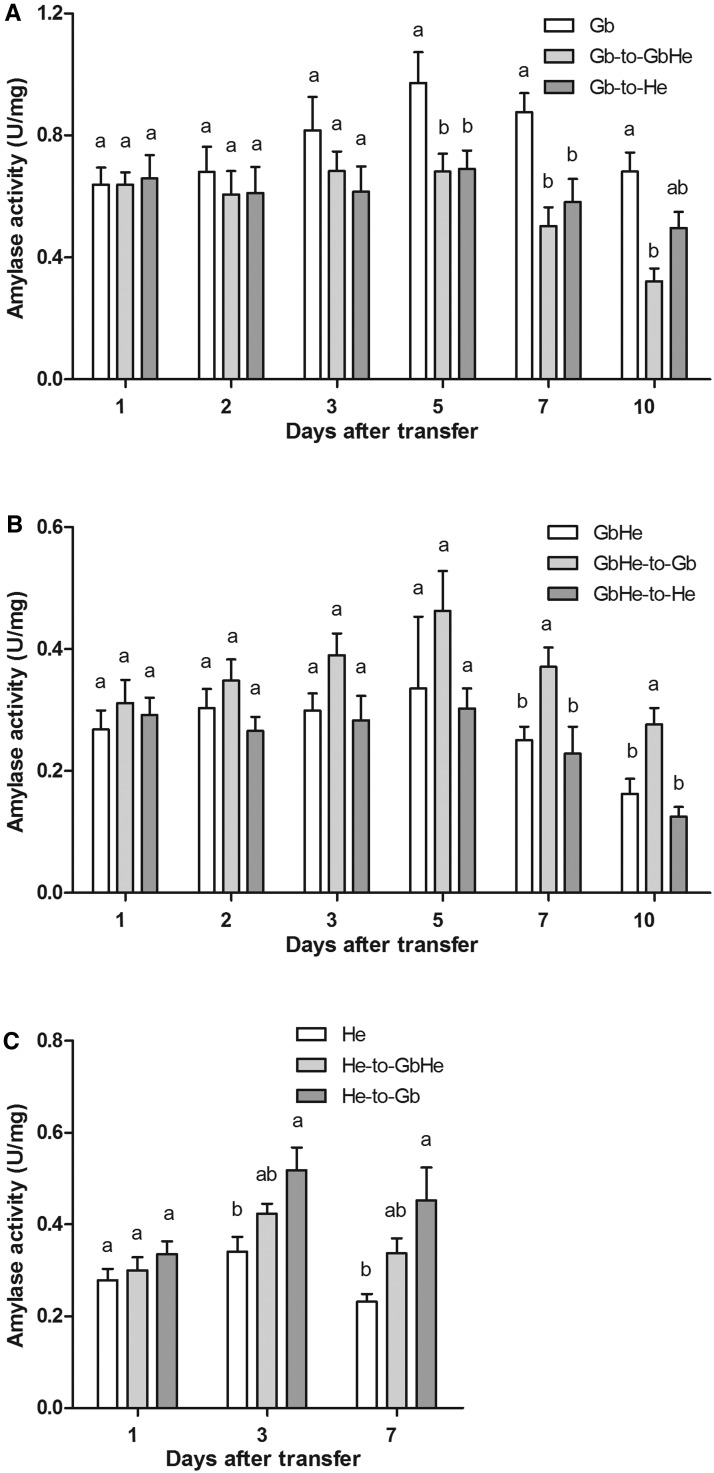
Amylase activity in *A. lucorum* at different times after food switching or in controls with one diet. (**A**) Gb, Gb transferred to GbHe (Gb-to-GbHe), Gb transferred to He (Gb-to-He); (**B**) GbHe, GbHe transferred to Gb (GbHe-to-Gb), GbHe transferred to He (GbHe-to-He); (**C**) He, He transferred to GbHe (He-to-GbHe), He transferred to Gb (He-to-Gb). Different letters by means for the same switching time among the various food switching treatments indicate significant differences at *P < *0.05 in a one-way ANOVA.

The activities of amylase changed distinctly with food switching (Fig. 3). When *A. lucorum* adults were transferred from Gb to He or GbHe, amylase activities tended to decline with increasing time after the switch (Fig. 3A). The decline of enzyme activity started at 2 d after transfer to GbHe or He. Five, 7, and 10 days after transfer, amylase activities on control of Gb were significantly higher than on transferred treatments of Gb-to-GbHe and Gb-to-He (for 5 d after: *F*_2,11 _=_ _3.98, *P *= 0.049; for 7 d after: *F*_2,8 _=_ _7.47, *P *= 0.015; for 10 d after: *F*_2,10 _=_ _10.76, *P *= 0.003).

Compared with the activity for the GbHe controls, amylase activity increased in the GbHe-to-Gb test, but did not change in the GbHe-to-He test (Fig. 3B). With increasing time after the transfer, amylase activity significantly differed among the three treatments (for 7 d after: *F*_2,8 _=_ _4.69, *P *= 0.045; for 10 d after: *F*_2,11 _=_ _9.41, *P *= 0.004).

Amylase activities increased starting by 1 d after the transfer from He to either Gb or GbHe (Fig. 3C). At 3 and 7 d after the transfer, amylase activity was higher on He-to-Gb than on He and He-to-GbHe (for 3 d after: *F*_2,7 _=_ _4.99, *P *= 0.045; for 7 d after: *F*_2,7 _=_ _4.24, *P *= 0.042).

### Effects of Food Switching on Trypsin-Like Enzyme Activities

**Fig. 4.**
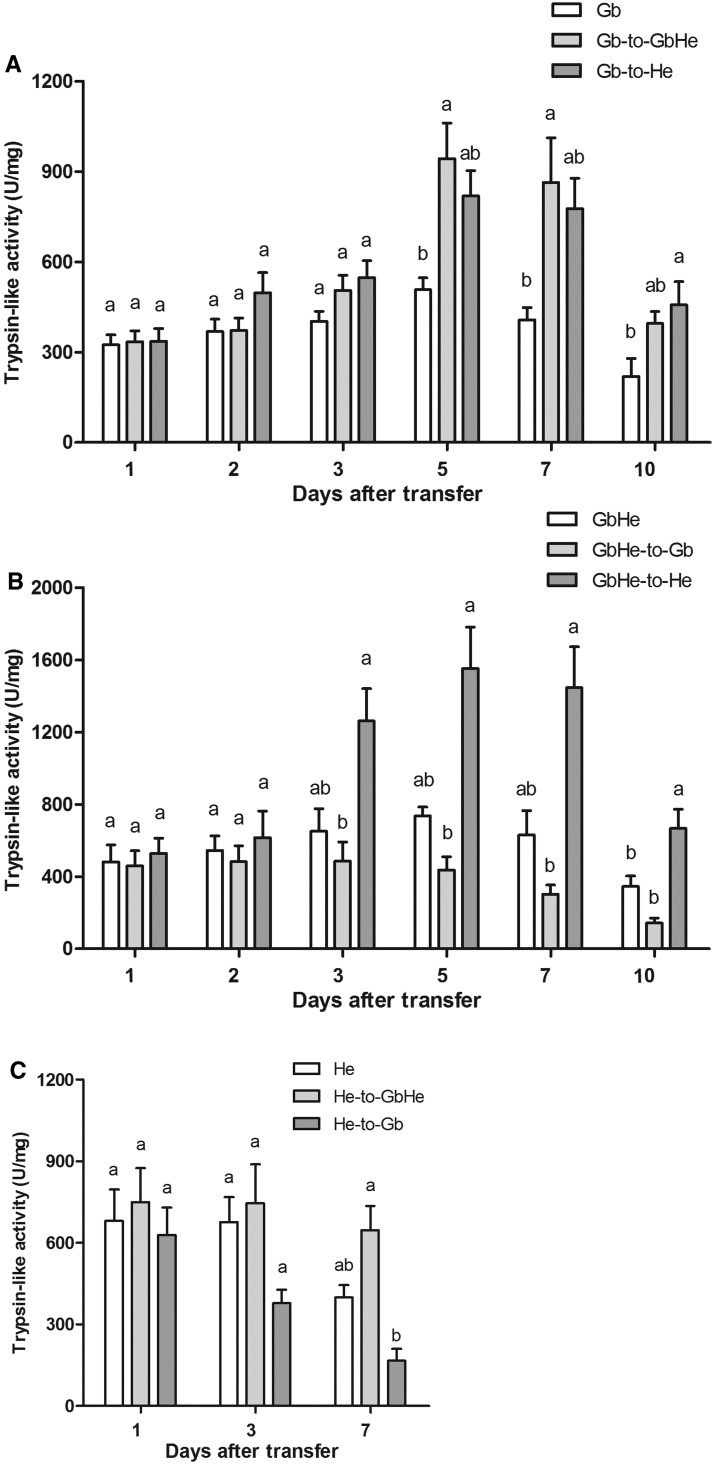
Trypsin-like enzyme activities in *A. lucorum* at different times after food switching or in controls with one diet. (**A**) Gb, Gb transferred to GbHe (Gb-to-GbHe), Gb transferred to He (Gb-to-He); (**B**) GbHe, GbHe transferred to Gb (GbHe-to-Gb), GbHe transferred to He (GbHe-to-He); (**C**) He, He transferred to GbHe (He-to-GbHe), He transferred to Gb (He-to-Gb). Different letters by means for the same switching time among the various food switching treatments indicate significant differences at *P < *0.05 in a one-way ANOVA.

In general, trypsin-like enzyme activities in *A. lucorum* on the Gb-to-GbHe and Gb-to-He treatments increased with transfer time compared with the Gb control (Fig. 4A). Significant differences were detected at 5, 7 and 10 d after the diet transfer among the three treatments (for 5 d after transfer: *F*_2,10 _=_ _8.04, *P *= 0.008; for 7 d after: *F*_2,8 _=_ _4.89, *P *= 0.046; for 10 d after: *F*_2,8 _=_ _4.63, *P *= 0.043). At 7 d after the transfer, the activities for the Gb-to-GbHe and Gb-to-He treatments were 864.02 ± 148.75 and 777.01 ± 101.01 U/mg protein, 2.12 and 1.91 times higher than for the Gb control.

Trypsin-like enzyme activities increased in the GbHe-to-He treatment, but decreased in GbHe-to-Gb treatment compared with the GbHe control (Fig. 4B). The activities of this digestive enzyme differed significantly among the three treatments at 3, 5, 7, and 10 d after transfer (for 3 d after: *F*_2,8 _=_ _8.52, *P *= 0.010; for 5 d after: *F*_2,8 _=_ _15.03, *P *= 0.002; for 7 d after: *F*_2,8 _=_ _14.54, *P *= 0.002; for 10 d after: *F*_2,9 _=_ _13.77, *P *= 0.002).

For the transfer from He to the other two food items, trypsin-like enzyme activities increased in the He-to-GbHe test but decreased in the He-to-Gb (Fig. 4C). The activities differed significantly among the three treatments at 7 dafter transfer (*F*_2,7 _=_ _16.65, *P *= 0.002).

### Effects of Food Switching on Chymotrypsin-Like Enzyme Activities

**Fig. 5.**
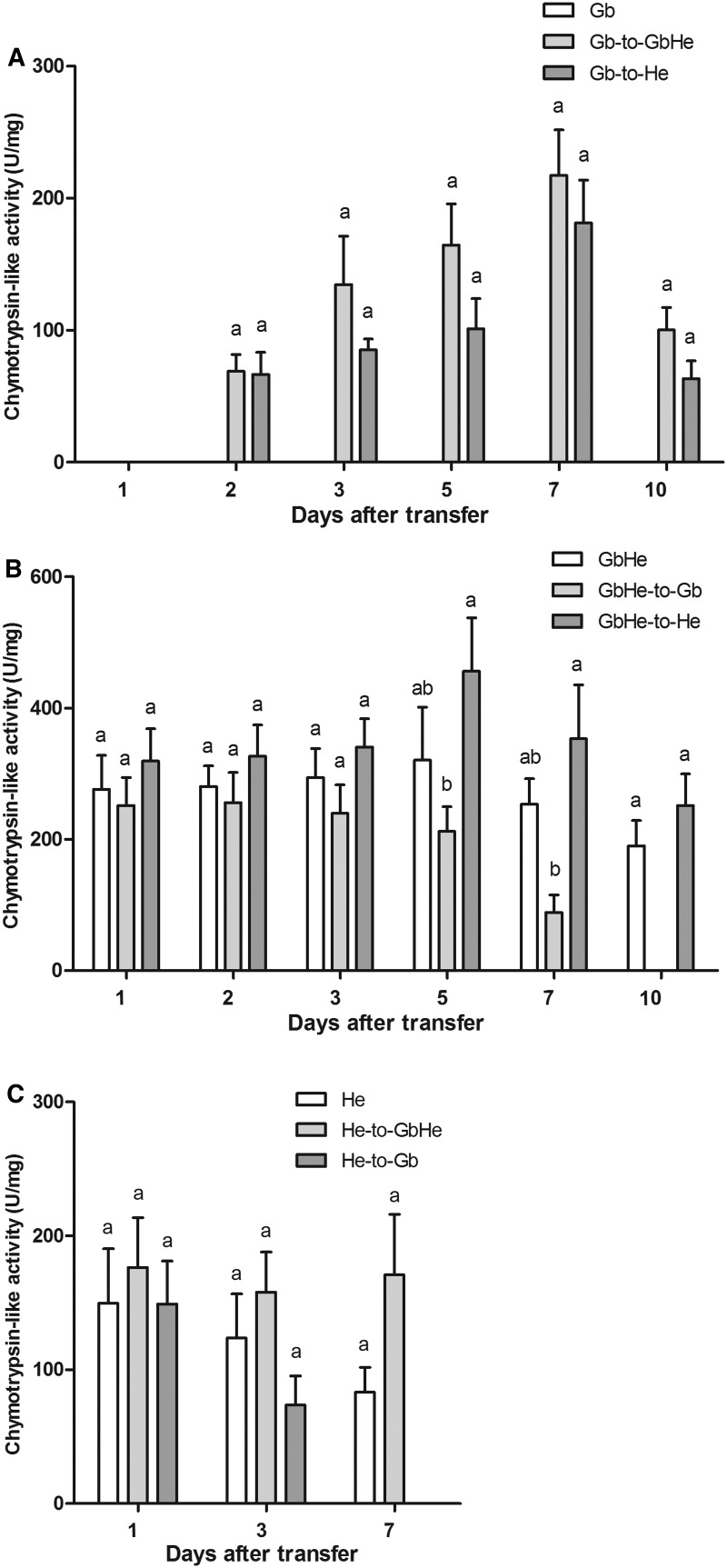
Chymotrypsin-like enzyme activities in *A. lucorum* at different times after food switching or in controls with one diet. (**A**) Gb, Gb transferred to GbHe (Gb-to-GbHe), Gb transferred to He (Gb-to-He); (**B**) GbHe, GbHe transferred to Gb (GbHe-to-Gb), GbHe transferred to He (GbHe-to-He); (**C**) He, He transferred to GbHe (He-to-GbHe), He transferred to Gb (He-to-Gb). Different letters by means for the same switching time among the various food switching treatments indicate significant differences at *P < *0.05 in a one-way ANOVA.

Chymotrypsin-like enzyme activity was not detected in insects feeding on Gb until 2 d after the transfer from Gb to GbHe or He (Fig. 5A). Chymotrypsin-like enzyme activities in the Gb-to-GbHe test did not differ significantly from the control (all *P *> 0.05).

Chymotrypsin-like enzyme activities increased in the GbHe-to-He treatment but decreased in the GbHe-to-Gb compared with the GbHe control (Fig. 5B). The activity differed significantly among the three treatments at 5 and 7 d after transfer (for 5 d after: *F*_2,9 _=_ _4.08, *P *= 0.046; for 7 d after: *F*_2,8 _=_ _5.82, *P *= 0.028). At 10 d after the transfer, the substrate for the chymotrypsin-like enzyme was not hydrolyzed by the enzyme sample from the bugs in the GbHe-to-Gb test. No significant difference was found between the GbHe and GbHe-to-He tests at 10 d after transfer (*F*_1,5 _= _ _0.87, *P *= 0.394).

When *A. lucorum* were transferred from He to the other two food items, chymotrypsin-like enzyme activity increased in the He-to-GbHe test but decreased in the He-to-Gb (Fig. 5C). However, no significant difference was observed between the He and He-to-GbHe tests at 1, 3, and 7 d after transfer (*F*_1,4 _=_ _3.21, *P *= 0.148). At 7 d after transfer, no substrate was hydrolyzed by the enzyme sample from the He-to-Gb treatment.

## Discussion

The composition and activity of digestive enzymes can reflect the consumer’s ability to use plant or animal food sources (Zeng and Cohen 2000, Tierno de Figueroa et al. 2011). Here, we detected amylase and proteases in *A. lucorum*. This species had amylase activity, especially in the salivary gland complex, which could indicate that it may extraorally digest and liquefy plant food sources. Amylase activity in the salivary glands and midguts of other phytophagous heteropterans has also been reported (Mehrabadi and Bandani 2009, Ravan et al. 2009). For instance, salivary gland secretions of the phytophagous bug *L. hesperus*, have amylase activity (Zeng and Cohen 2000). Amylase activity is not expected, however, in strictly zoophagous insects. David and Boyd (2003) found no amylase activity in the salivary gland complex of the zoophagous mirid bug *Deraeocoris nigritulus* (Heteroptera: Miridae), indicating that this species is not a strict phytophage.

*Apolygus* *lucorum* had strong protease activity, especially in the salivary gland complex, which indicates it can use extraoral digestion to use structural proteins in animal food sources. Zeng and Cohen (2001) found protease activity in the salivary gland complex of omnivorous hemipteroid *L. hesperus*. Variations in protease activity were also found in the predator *Podisus maculiventris* (Say) (Hemiptera: Pentatomidae) (Pascual-Ruíz et al. 2009). Cohen and Wheeler (1998) showed that digestive proteases were absent or low in the salivary gland complex of the strictly phytophagous mirid *P.lineatus* (Heteroptera: Miridae).

The presence of trypsin-like enzyme in the salivary gland complex and midgut of *A. lucorum* further affirmed the ability of this mirid to extraorally digest proteins from animal food sources. A previous study showed strong trypsin-like enzyme activity in the assassin bug *Zelus renardii* Kolenati (Hemiptera: Reduviidae) (Cohen 1993). We also found evidence for chymotrypsin-like enzyme activity in the midgut of *A. lucorum*, but none in the salivary glands. The presence of trypsin-like enzyme activity but the absence of chymotrypsin-like enzyme activities in the salivary glands of *A. lucorum* may be a result of its zoophagous feeding strategy or digestive plasticity of this omnivorous insect (Boyd et al. 2002, Wright et al. 2006).

The low amylase activity and high protease activity may be a common trait in hemipteran mirids, especially omnivorous bugs, which be both phytophagous and zoophagous. In *A. lucorum*, amylase and trypsin-like enzyme activities in the salivary gland complex of females at 10 d of age were 0.97 ± 0.10 U/mg and 507.61 ± 39.82 U/mg, respectively. In predatory bug, *Podisus nigrispinus*, amylase and cathepsin (the main protease in this bug) activities were 0.02 ± 0.004 U/mg and 3800 ± 300 U/mg (Fialho et al. 2012), respectively.

The activities of digestive enzymes varied in different sexes at different ages in *A. lucorum*. Higher activities in the middle stage (10 and 15 d) of female adults tested were found in the salivary gland complex and midgut of *A. lucorum*. These may indicate that the digestive enzymes appear to have crucial roles in reproduction. During the period, from preoviposition to the peak of oviposition of *A. lucorum*, high nutrient levels are required (Yuan et al. 2013).

*Apolygus* *lucorum*, as an omnivorous insect, feeds on a variety of crop plants and small insects. Such omnivore might involve major shifts and flexibility in metabolism to cope with a diverse diet. In the present study, the plant diet led to higher amylase activities than did the animal diet. High amylase activity in the plant-fed adults demonstrated that this mirid species can utilize plant material for nutrition. In adults fed a combination of plant and animal food or only an animal source, protease (trypsin-like and chymotrypsin-like enzymes) activity was higher than in adults fed only plants. High protease activity in the adults fed on the combination food and on the animal food indicated that this mirid could also use animal material for nutrition. Food sources also affect the activity of digestive enzymes of other insects. Digestive enzymes of *Gryllus bimaculatus* (Orthoptera: Gryllidae) released in response to nutrients in the food (Woodring et al. 2009). Sugars in food increase the release of amylase in *G. bimaculatus*, and low concentrations of protein increase trypsin release. Larvae of the fall armyworm *Spodoptera frugiperda* (J.E. Smith) (Lepidoptera: Noctuidae) adjust amylase activity for carbohydrate ingestion, and indeed, amylase activity is significantly higher in fed carbohydrate larvae than in unfed larvae (Lwalaba et al. 2010).

Results of the food switching test showed that amylase and protease activities changed significantly in the salivary gland complex of *A. lucorum* by 5 or 7 d after the transfer to a different diet. Thus, within a certain time, the activities of the digestive enzymes in *A. lucorum* can change in response to changes in nutrient conditions of the diet. Other insects can respond similarly (Zeng and Cohen 2001, Yan et al. 2011). For example, different host plant sources can result in differing salivary digestive enzyme activities in *B. tabaci* (Yan et al. 2011). Different prey also induce variations in protease activity in the predator *P. maculiventris* (Pascual-Ruíz et al. 2009).

In summary, the presence of amylase and protease in the salivary gland complex and midgut of *A. lucorum* indicates that this species can be both phytophagous and zoophagous. The activities of these enzymes are affected by sex, age, and food source. Such findings reveal the relative levels of phytophagy and zoophagy in both sexes of *A. lucorum* at different ages. In addition, the results of this study will be useful for laboratory rearing and for further elucidating the feeding mechanism of *A. lucorum*.
